# Relationship between diet quality and depression among Iranian older adults in Tehran

**DOI:** 10.1186/s12877-022-03380-1

**Published:** 2022-08-26

**Authors:** Maedeh Chegini, Pedram Shirani, Nasrin Omidvar, Hassan Eini-Zinab, Fatemeh Pour-Ebrahim, Arezoo Rezazadeh

**Affiliations:** Department of Community Nutrition, National Nutrition and Food Technology Research Institute, School of Nutrition and Food Technology, Shahid Beheshti University of Medical Sciences, Tehran, Iran

**Keywords:** Diet, Healthy Eating Index, Depressive Disorder, Aged, Tehran

## Abstract

**Background:**

Depression is one of the prevalent mental disorders that is common in older ages. Evidence suggests that dietary intake status may potentially be associated with depression. However, this association has not been well studied in older adults, and the nature of the association remains unclear. This study aimed to investigate the association between diet quality and depression in free-living older adults living in Tehran city.

**Methods:**

This cross-sectional study was conducted on 583 older adults (279 men and 304 women) aged 60 to 80 years living in Tehran city, selected by the systematic cluster sampling method. Individuals' dietary intake was determined by two non-consecutive 24-h dietary recalls, and diet quality was assessed calculating score of Healthy Eating Index-2015 (HEI-2015). The validated short form of the Geriatric Depression Scale (GDS-11) was used to assess depression status. A logistic regression model was used to assess the chance of depression across tertiles of HEI-2015 score.

**Results:**

The point prevalence of depression was 22.5%, which was significantly higher in women (91 (69.5%) women vs. 40 (30.5%) men). A higher percentage of seniors at the higher tertile of HEI score were women, belonged to Fars ethnicity and had a higher score of activity of daily living (ADL). After adjustment for all potential confounders, the trend of depression chance was significantly decreased in those with higher HEI-2015 scores (*p*-for trend < 0.05).

**Conclusion:**

Higher quality of diet was associated with a lower possibility of depression in the studied participants. Further studies are required to investigate the possible causal relationship.

**Supplementary Information:**

The online version contains supplementary material available at 10.1186/s12877-022-03380-1.

## Background

Ageing is an inevitable physiologic process of every living organism, that is associated with decreased homeostatic control, the ability to reconcile to environmental factors and the capacity for stress response [1, 2]. The number of older adults is increasing worldwide [3]; that was reached 962 million in 2017 and it is estimated to be double by 2050 [4]. Global ageing is associated with economic, social, and health challenges [2–5]. According to the Iran`s national document of elderly, the age of 60 was defined as the beginning of old age [6]. Based on the National Population & Housing Census of Iran, more than 9% of the Iranian population was over 60 in 2016, and it is expected to reach around 22% in 2050 [7, 8]. The Middle East is also following a similar trend [9]. The number of older adults is swiftly increasing in Egypt, Jordan, and Turkey, and the percentage of the populations aged over 65 in these countries varied from less than 4 percent to 8.5 percent in 2018 [10, 11].

Depression is a prevalent psychiatric disorder that is described by depressive mood, loss of motivation, lack of physical energy, inability to feel pleasure, disturbed sleep, disability and worthlessness, and ultimately a reduced sense of concentration [12]. About 5 million seniors are suffering from geriatric depression globally, and might never be recognized or poorly treated [13]. As a result, poor functioning, increased perception of poor health, increased demand for medical services could increase the rate of morbidity and mortality in this vulnerable groups [14, 15]. Physiologic changes, as well as social and environmental factors such as poor socioeconomic status, social isolation, reduced independence, loneliness, and various chronic diseases, all contribute to the high prevalence of depression in older adults [16–18]. However, lifestyle factors, particularly nutritional status have recently been addressed in the etiology of depression and they are regarded as the most modifiable component in the prevention or control of the disease [19, 20].

There is some evidence that diet quality is inversely related to depression in older adults. Nevertheless, the relationship between depression and nutritional status is still unclear [5, 21]. Findings from a meta-analysis of longitudinal and cross-sectional studies have shown a significant inverse association between diet quality and depressive outcomes [5]. However, there are limited data regarding the association between nutritional quality indexes and depression in older adults in the Middle East, where has different dietary habits and the limited investigations on social and biological factors led to different conclusions about the causal relationship between diet quality and depression. Therefore, the aim of the current study was to examine the association between diet quality and depression in a sample of urban free-living older adults in Tehran.

## Methods

### Study design and sampling

The current cross-sectional study is part of a comprehensive study entitled “situation analysis of free-living elders' Lifestyle (with Emphasis on Nutrition)” that was undertaken on 583 older adults (279 men and 304 women) aged 60 to 80 years old living in Tehran city [22]. Due to the varied socio-economic status of the residents of different districts, all geographical zones of Tehran (north, south, east, west, and center) were included, and 11 municipal districts were systematically selected across the zones. Considering population weight, the number of seniors required for sampling in each area was determined. In each district, 60% of the subjects were selected from a random health center, and the rest were selected from community centers (*Saraye Mahalleh*) [30%], and mosques [10%] close to the selected health centers. The inclusion criteria of the subjects were being a free-living older adult (60–80 years old) in Tehran, having Iranian citizenship with the ability of logical communication, and having no serious medical condition, including cancer, ESRD, severe metabolic and cognitive disorders, e.g., Alzheimer's and Parkinson's disease.

Data collection was carried out by nutrition experts via face-to-face interviews. To avoid interviewer bias, they received training for data collection in a one-day workshop.

### Assessment of dietary intakes

The dietary intake of the subjects was assessed by completing two non-consecutive 24-h recall questionnaires (one week day and one weekend day) via the multiple-pass method [23], which has been applied in previous studies in Iranian older adults [24–27]. The first recall was obtained in person by a nutritionist, and the second was by telephone. During the interviews, participants were asked to recall food and drinks they had consumed during the past 24 h. Afterward, the information about two 24-h recalls was reviewed and the reported home values were converted to grams using the Iranians’ Home Scale Guide [28]. Finally, the United States Department of Agriculture food composition table [FCT] (USDA, Release 11, 1994) along with the Iran FCT (which is only for raw food items) [29] were used to calculate energy and nutrient intake values.

### Assessment of adherence to Healthy Eating Index 2015 (HEI-2015)

The healthy Eating Index 2015 (HEI-2015) is the latest version of the HEI which is used to determine nutritional quality by calculating energy-adjusted intakes of its components (Total Fruits, Whole Fruits, Total Vegetables, Greens and Beans, Whole Grains, Dairy, Total Protein Foods, Seafood and Plant Proteins, Unsaturated Fat/Saturated Fat, Refined Grains, Sodium, Added Sugars, and Saturated Fats). Each HEI component was graded from 0 to 10, and the total HEI-2015 score was computed by summing up the scores of its components, which ranged from zero to 100. Afterward, the total HEI-2015 score was categorized, as less than 50 showing poor nutritional status, 50 to 80 reflecting that the quality of diet needed to be improved, and above 80 was considered a favorable status. The HEI-2015 and HEI-2010 are so similar, except for two items, including saturated fat and added sugars, that were considered exclusively in the new version [30]. Detailed information on the HEI-2015 content and construct, as well as criterion validity and reliability, are available elsewhere [31]. The face validity of HEI-2015 was assessed by an expert panel and all items of the original index were approved for the studied population.

### Assessment of outcome

Depression status was assessed by a short form of the Geriatric Depression Scale (GDS) containing 11 closed-ended questions [31]. GDS is a brief questionnaire used as a screening test for depression in older adults. The maximum depression score was 11. Scores of 6 or more were considered the presence of depression, and scores of 0–5 were defined as normal. The validity and reliability of the Geriatric Depression Scale (GDS-11) were previously evaluated in Iranian older adults and represented an acceptable sensitivity (0.9) and specificity (0.83) with GDS-15 [32, 33].

### Assessment of covariates

The demographic and socioeconomic status was assessed by a questionnaire used in previous studies [34], where the variables were defined as follows: gender (male, female), age group (60–65, 65–74, 74–80 years), marital status (single/widow/divorced, married), ethnicity (Fars, Azeri, Gilak and Mazani, others), educational level (illiterate/less than a diploma, high school diploma, academic education), living status (alone or with family/nurse), house possession (yes, no), monthly household income (million IRR), receiving social services or food aid (yes, no), and being under the coverage of insurance or supplemental insurance (yes, no).

Anthropometric measures included weight measured using a portable digital scale (803 Seca) with an accuracy of 100 g and height, waist circumference, mid-arm circumference (distance between the Acromion and the Olecranon appendices), and calf circumference (the thickest part of the calf without clothing) measured using a tape measure with an accuracy of 1 mm by standard methods [35]; BMI was calculated by dividing weight (kg) by the square of height (m^2^).

The physical function of individuals was assessed using the 7-item questionnaire of Activity of Daily Living (ADL) and the 9-item questionnaire of Instrumental Activity of Daily Living (IADL). According to the ADL questionnaire, seniors with scores of 11–14 were considered independent, and in terms of the IADL questionnaire, participants with scores of 14 or higher were deemed independent. More details about the content and scoring procedure of these questionnaires have been previously described elsewhere [36]. The validity and reliability of these questionnaires in Iranian older adults have been assessed previously [36, 37].

Other lifestyle habits such as current smoking (yes, no) and participants’ health status, including medical history and medication, dietary supplement intake (yes, no), and sleep habits (regular, irregular) were obtained through a face-to-face interview.

### Data analysis

The data were analyzed using IBM SPSS (version 21.0). After data cleaning, the Kolmogorov–Smirnov test was used to determine if the distribution of variables is normal. Initially, the samples were described according to the subjects’ general characteristics as defined by descriptive tests (t-test, Chi-square, wherever suitable). Of the 583 participants, 511 had acceptable dietary intake data (two complete 24 h-recall in the defined cut-off of energy intake) were considered in the diet quality analysis. The HEI-2015 total score was categorized as tertiles and the characteristics of participants across the tertiles of the HEI-2015 score were assessed by a one-way ANOVA test for quantitative variables and a chi-square test for qualitative variables, and results were reported as mean ± SD and count (percentage) respectively.

In order to estimate the relationship between HEI-2015 and its components and depression, a binary logistic regression test was applied and the odds ratio and 95% confidence interval (CI) were reported in 3 models. First, demographic variables (age, sex, marital status), physical functioning (ADL, IADL), and energy intake were controlled. In the second model, additional adjustments were applied for socioeconomic factors (education, living status (alone or with family/nurse), house possession, household income, receiving social services or food aid, insurance and supplemental insurance coverage). In the final model, further adjustments were made for health status and behavioral confounders (gastrointestinal problems, oral problems, medication status, dietary supplement intake, smoking status, and sleep habits), anthropometric indices (weight, waist circumference, mid-arm circumference, and calf circumference), and obesity. In all sections, individuals in the first category of HEI-2015 were considered the reference group. Two-tailed *P* values < 0·05 were considered statistically significant. As a basis for the trend test, the HEI-2015 score was constructed from the categorized variable and placed into the model as a successive integer. Odds ratios of covariates in the 3 models are presented in supplementary tables (S1, S2 and S3).

## Results

Of the 583 subjects, 304 (52.1%) were women and 279 (47.8%) were men, with a mean ± SD age of 67.87 $\pm$ 5.86 years (men (69.54 $\pm$ 6.144) and women (66.34 $\pm$ 5.143)). The general characteristics of participants across GDS-SF scores are presented in Table 1. About a quarter (22.5%) of participants had depression. Higher percent of those detected with depression were females, unmarried, living alone, taking medications, with a lower educational degree (< 12 years), no paid job, and lower household income, had irregular sleep pattern and were more dependent based on IADL score and they had higher BMI and mid-arm circumference.

**Table 1:** General characteristics of participants across GDS-SF scores

Variables		Total n (%)	GDS-SF scores n (%)		*p*-value
			$<$**6** **(normal)**	$\ge$**6** **(with depression)**	
**Total n (%)**		583	452 (77.5)^a^	131 (22.5)^a^	
**Age (years)**	65 – 60	203 (34.8)	160 (35.4)	43 (32.8)	**0.340**
	74—65	268 (46.0)	211 (46.7)	57 (43.5)	
	80—74	112 (19.2)	81 (17.9)	31 (23.7)	
**Sex**	Male	279 (47.9)	239 (52.9)	40 (30.5)	**0.001 > **
	Female	304 (52.1)	213 (47.1)	91 (69.5)	
**Ethnicity**	Fars	328 (56.5)	254 (56.4)	74 (56.4)	**0.365**
	Azeri	126 (21.7)	95 (21.1)	31 (23.7)	
	Gilak and Mazani	37 (6.4)	26 (5.8)	11 (8.4)	
	Other ethnics	90 (15.5)	75 (16.7)	15 (11.5)	
**Education**	Illiterate/Primary/secondary school	392 (67.2)	285 (63.1)	107 (81.7)	**0.001 > **
	High school diploma	107 (18.4)	89 (19.7)	18 (13.7)	
	University degree	84 (14.4)	78 (17.3)	6 (4.6)	
**Marital status**	Single/widow/divorced	119 (20.4)	83 (18.4)	36 (27.5)	**0.027**
	Married	464 (79.6)	369 (81.6)	95 (72.5)	
**Living status**	Alone	68 (11.7)	47 (10.4)	21 (16.0)	**0.089**
	With family/nurse	515 (88.3)	405 (89.6)	110 (84.0)	
**Job status**	with income (paid job)	309 (53.0)	267 (59.1)	42 (32.1)	**0.001 > **
	With no income	274 (47.0)	185 (40.9)	89 (67.9)	
**Monthly household income (million IRR)**	< 10	96 (16.9)	48 (10.9)	48 (37.2)	**0.001**^**b**^** > **
	10 – 20	266 (46.7)	206 (46.8)	60 (46.5)	
	20 – 30	110 (19.3)	96 (21.8)	14 (10.9)	
	> 30	97 (17.0)	90 (20.5)	7 (5.4)	
**Smoking status**	Current smoker	46 (7.9)	38 (8.4)	8 (6.2)	**0.465**
**Sleep pattern**	Irregular	232 (40.0)	150 (33.3)	82 (63.1)	**0.001 > **
**Taking medication**	yes	505 (87.4)	381 (85.2)	124 (94.7)	**0.004**
**ADL**	Independent	534 (92.1)	407 (90.6)	127 (96.9)	**0.007**^**b**^
**IADL**	Independent	487 (84.5)	391 (87.3)	96 (75.0)	**0.003**^**b**^
**BMI (kg/m**^**2**^**)**^**c**^		28.3521 $\pm$ 4.69739	28.0975 $\pm$ 4.63335	29.2176 $\pm$ 4.82725	**0.017**
**Mid-arm circumference (mm)**^**c**^		30.4329 $\pm$ 3.61279	30.2393 $\pm$ 3.41241	31.0977 $\pm$ 4.17608	**0.017**
**Calf circumference (mm)**^**c**^		37.2672 $\pm$ 3.69475	37.1982 $\pm$ 3.50423	37.5054 $\pm$ 4.29493	**0.404**

χ2 test was used to analyze qualitative variables and the percentages are based on GDS-SF categories

^a^Percentages are related to the total number of studied population

^b^Fisher’s Exact test was used for this variable

^c^Obtained from ANOVA test and the results are reported as mean ± SD

*ADL* activities of daily living, *IADL* instrumental activities of daily living

For ADL and IADL, only independent groups were reported

Furthermore, in comparison with normal subjects, a higher percentage of depressed seniors were suffering from gastrointestinal (149 [33%] vs. 71 [54.2%]; *p*-value $<$ 0.001) or oral disorders (99 [21.9% vs. 51 (38.9%)]; *p*-value $<$ 0.001) (data not shown in the table).

Table 2 describes general characteristics of the target population according to tertiles (T) of HEI-2015. The majority of seniors (76.5%) were at the middle tertile, 19.5% were at the highest and 4% were at the lowest tertile of HEI-2015. Participants in the highest tertile of HEI-2015 were more likely to be women, from Fars ethnicity, and independent according to the ADL physical function questionnaire. There were no significant differences in other characteristics of studied seniors across the categories of HEI-2015.

**Table 2:** General characteristics of target population across tertiles (T) of Healthy Eating Index-2015 (HEI-2015)

Variables		Total n(%)	Tertiles of HEI-2015			*p*-value
			**T**_**1**_	**T**_**2**_	**T**_**3**_	
**Total n(%)**		511	20 (3.9)^a^	391 (76.5)^a^	100 (19.6)^a^	
**Age (years)**	65—60	178 (34.8)	30.0)) 6	130 (33.2)	42 (42.0)	**0.377**^**b**^
	74—65	240 (47.0)	12 (60.0)	185 (47.3)	43 (43.0)	
	80- 74	93 (18.2)	2 (10.0)	76 (19.4)	15 (15.0)	
**sex**	Male	261 (51.1)	16 (80.0)	199 (50.9)	46 (46.0)	**0.020**^**b**^
	Female	250 (48.9)	4 (20.0)	192 (49.1)	54 (54.0)	
**Ethnicity**	Fars	299 (58.7)	55.0)) 11	(56.2) 219	(69.7) 69	**0.047**
	Other ethnic groups	210 (41.3)	(45.0) 9	43.8)) 171	(30.3) 30	
**Education**	Under-diploma	337 (65.9)	12 (60.0)	261 (66.8)	64 (64.0)	**0.780**^**b**^
	Diploma	93 (18.2)	3 (15.0)	70 (17.9)	20 (20.0)	
	University degree	81 (15.9)	5 (25.0)	60 (15.3)	16 (16.0)	
**Marital status**	Single/widow/divorced	95 (18.6)	0 (0.0)	74 (18.9)	21 (21.0)	**0.054**^**b**^
	Married	416 (81.4)	(100.0) 20	317 (81.1)	79 (79.0)	
**Living status**	Alone	54 (10.6)	2 (10.0)	42 (10.7)	10 (10.0)	**1.000**^**b**^
	With family/nurse	457 (89.4)	18 (90.0)	349 (89.3)	90 (90.0)	
**Job status**	with income (paid job)	285 (55.8)	16 (80.0)	214 (54.7)	55 (55.0)	**0.084**^**b**^
	With no income	226 (44.2)	4 (20.0)	177 (45.3)	45 (45.0)	
**Monthly household income(million IRR)**	< 10	80 (16.1)	2 (10.0)	59 (15.5)	19 (19.4)	**0.306**^**b**^
	10 – 20	219 (44.0)	7 (35.0)	178 (46.8)	34 (34.7)	
	20 – 30	107 (21.5)	6 (30.0)	77 (20.3)	24 (24.5)	
	> 30	92 (18.5)	5 (25.0)	66 (17.4)	21 (21.5)	
**Smoking status**	Current smoker	44 (8.6)	1 (5.0)	33 (8.5)	10 (10.0)	**0.815**^**b**^
**Sleep pattern**	Irregular	195 (38.4)	5 (25.0)	157 (40.3)	33 (33.7)	**0.222**
**Taking medication**	yes	439 (86.2)	16 (80.0)	341 (87.7)	82 (82.0)	**0.209**^**b**^
**ADL**	Independent	476 (93.7)	8 (40.0)	369 (94.9)	99 (100.0)	**0.001**^**b**^
**IADL**	Independent	423 (83.8)	17 (85.0)	323 (83.5)	83 (84.7)	**0.823**^**b**^
**BMI (kg/m**^**2**^**)**^**c**^		28.4019 $\pm$ 4.65481	27.6986 $\pm$ 4.03770	28.2945 $\pm$ 4.68932	28.9602 $\pm$ 4.64570	**0.351**
**Mid-arm circumference (cm)**^**c**^		30.4768 $\pm$ 3.57469	39.4700 $\pm$ 5.43895	30.4715 $\pm$ 3.46207	30.6990 $\pm$ 3.55847	**0.373**
**Calf circumference (cm)**^**c**^		37.3854 $\pm$ 3.72989	35.8789 $\pm$ 5.88270	37.4461 $\pm$ 3.62947	37.4360 $\pm$ 3.58703	**0.200**

χ2 test was used to analyze qualitative variables and the results are reported as n (%). Percentages are based on HEI categories

^a^Percentages are related to the total number of studied population

^b^Fisher’s Exact test was used for this variable

^c^Obtained from ANOVA test and the results are reported as mean ± SD

*ADL* activities of daily living, *IADL* instrumental activities of daily living, *BMI* body mass index

For ADL and IADL, only independent groups were reported

The association between depression and diet quality of subjects was shown in Table 3. Individuals who were in the highest tertile of HEI had a lower chance of depression in comparison with those in the lower tertiles, but the *p*-value was not significant in the crude and adjusted models. However, the trend of the depression chance was statistically significant for the last model (OR = 0.176, CI 95%: 0.020 – 1.524, *p* = 0.115; *p*-for-trend < 0.05). The relationship between depression and HEI-2015 components is summarized in Table 4. Even after adjustment for confounders, no significant association was observed between HEI-2015 components’ scores and depression.

**Table 3:** Odds ratios of depression across tertiles of Healthy Eating Index-2015 (HEI-2015) in the studied older adults in Tehran

		**Tertiles of HEI-2015**					***P-trend***
		**T**_**1**_	**T**_**2**_		**T**_**3**_		
		***OR***	***OR (95% CI)***	***P*-value**	***OR (95% CI)***	***P*-value**	
**Depression**	**Crude**	1.000 (ref.)	1.670 (0.478 – 5.828)	0.421	1.000 (0.261 – 3.836)	1.000	**0.261**
	**Model 1**	1.000 (ref.)	0.690 (0.163 – 2.916)	0.614	0.418 (0.089 – 1.969)	0.270	**0.108**
	**Model 2**	1.000 (ref.)	0.558 (0.123 – 2.532)	0.450	0.314 (0.061 – 1.613)	0.165	**0.073**
	**Model 3**	1.000	0.429 (0.057 – 3.205)	0.409	0.176 (0.020 – 1.524)	0.115	0.021*

Depression was considered as a dependent binary variable and HEI as the independent variable

Model 1: adjusted for physical function (ADL and IADL), energy intake and demographic indicators (age, sex, marital status)

Model 2: further adjustments for socio-economic variables (education, status of living (alone or with family/nurse), house possession, household income, receiving social services or food aids, insurance and supplemental insurance coverage)

Model 3: more adjustments for health status and behavioral confounders (gastrointestinal problems, oral problems, medication status, dietary supplements, smoking status and sleep habits), anthropometric indices (weight, waist circumference, mid-arm circumference and calf circumference) and obesity

The first tertile of Healthy Eating Index 2015 is considered as the reference group

*P-value<0.05 was considered statistically significant

**Table 4:** Odds ratios of depression across Healthy Eating Index-2015 (HEI-2015) components in the studied older adults in Tehran

HEI-2015 Components scores		Depression		
		***OR***	***95% CI***	***P*-value**
**Total Fruits**	**Crude**	0.972	0.837 – 1.130	**0.715**
	**Model 1**	0.930	0.789 – 1.097	**0.390**
	**Model 2**	0.934	0.780 – 1.119	**0.460**
	**Model 3**	0.895	0.723 – 1.107	**0.306**
**Whole Fruits**	**Crude**	0.995	0.828 – 1.196	**0.958**
	**Model 1**	0.940	0.769 – 1.149	**0.546**
	**Model 2**	0.929	0.743 – 1.162	**0.519**
	**Model 3**	0.878	0.674 – 1.144	**0.335**
**Total Vegetables**	**Crude**	1.118	0.948 – 1.319	**0.185**
	**Model 1**	1.147	0.957 – 1.376	**0.138**
	**Model 2**	1.258	1.031 – 1.535	**0.024**
	**Model 3**	1.255	0.992 – 1.587	**0.060**
**Greens and Beans**	**Crude**	1.110	0.984 – 1.251	**0.086**
	**Model 1**	1.124	0.987 – 1.280	**0.077**
	**Model 2**	1.114	0.971 – 1.278	**0.124**
	**Model 3**	1.118	0.950 – 1.315	**0.181**
**Whole Grains**	**Crude**	0.974	0.924 – 1.027	**0.337**
	**Model 1**	0.976	0.921 – 1.035	**0.414**
	**Model 2**	0.989	0.929 – 1.053	**0.728**
	**Model 3**	0.991	0.920 – 1.067	**0.802**
**Dairy**	**Crude**	1.013	0.940 – 1.093	**0.727**
	**Model 1**	1.021	0.938 – 1.110	**0.631**
	**Model 2**	1.022	0.933 – 1.120	**0.639**
	**Model 3**	1.035	0.932 – 1.149	**0.524**
**Total Protein Foods**	**Crude**	1.135	0.928 – 1.388	**0.216**
	**Model 1**	1.137	0.917 – 1.408	**0.241**
	**Model 2**	1.109	0.879 – 1.400	**0.383**
	**Model 3**	1.167	0.886 – 1.536	**0.271**
**Seafood and Plant Proteins**	**Crude**	0.953	0.855 – 1.062	**0.381**
	**Model 1**	0.990	0.878 – 1.117	**0.876**
	**Model 2**	0.998	0.877 – 1.137	**0.982**
	**Model 3**	0.962	0.824 – 1.123	**0.623**
**Unsaturated fat/saturated fat**	**Crude**	1.013	0.896 – 1.144	**0.841**
	**Model 1**	1.000	0.870 – 1.148	**0.998**
	**Model 2**	0.999	0.862 – 1.157	**0.990**
	**Model 3**	1.030	0.867 – 1.223	**0.737**
**Refined Grains**	**Crude**	0.966	0.909 – 1.027	**0.272**
	**Model 1**	0.950	0.887 – 1.017	**0.141**
	**Model 2**	0.977	0.907 – 1.052	**0.529**
	**Model 3**	0.999	0.916 – 1.090	**0.984**
**Sodium**	**Crude**	1.042	0.987 – 1.101	**0.135**
	**Model 1**	1.017	0.957 – 1.081	**0.584**
	**Model 2**	0.992	0.930 – 1.060	**0.822**
	**Model 3**	0.946	0.874 – 1.023	**0.166**
**Added Sugars**	**Crude**	1.055	0.927 – 1.201	**0.415**
	**Model 1**	1.073	0.927 – 1.242	**0.342**
	**Model 2**	1.120	0.947 – 1.325	**0.186**
	**Model 3**	1.192	0.973 – 1.461	**0.090**
**Saturated Fats**	**Crude**	1.037	0.948 – 1.134	**0.427**
	**Model 1**	1.019	0.924 – 1.124	**0.701**
	**Model 2**	0.987	0.890 – 1.094	**0.802**
	**Model 3**	0.966	0.856 – 1.090	**0.572**

Depression was considered as a dependent binary variable and HEI-2015 components as the independent variable

Model 1: adjusted for physical function (ADL and IADL), energy intake and demographic indicators (age, sex, marital status). Model 2: further adjustments for socio-economic variables (education, status of living (alone or with family/nurse), house possession, household income, receiving social services or food aids, insurance and supplemental insurance coverage). Model 3: more adjustments for health status and behavioral confounders (gastrointestinal problems, oral problems, medication status, dietary supplements, smoking status and sleep habits), anthropometric indices (weight, waist circumference, mid-arm circumference and calf circumference) and obesity

The first tertile of Healthy Eating Index 2015 is considered as the reference group

## Discussion

This study investigated associations between diet quality and geriatric depression in Iranian older adults for the first time. After adjustment for possible confounders, participants who had a higher HEI score displayed a lower chance of depression. Our findings indicated a depression prevalence of 22.5%, which was lower than previous reports conducted in Iranian older adults [38, 39]. According to Taheri Tanjanai et al. [40], the prevalence of depression among 1350 older adults selected from five provinces of Iran was 36.7%. Also, the prevalence was notably lower than similar research in some middle-east and developing countries. As an instance, 83.75% of senior participants in Bangladesh displayed different degrees of depression symptoms [41]. Furthermore, another study of 770 senior citizens in Turkey showed a depression prevalence of 51.8% according to GDS-15 [42]. Wide variance in the instruments used for measuring depression may explain part of these significant differences. In the present study, depression frequency was higher in old-age women than men. As with our finding, many researchers have discovered that women are more susceptible to depression than men [43–45]. This correlation may be explained by social gender inequalities in the studied population, and a reduction in hormone secretion after menopause, which may lead to depression development [42, 46]. We also observed lower educational levels and socioeconomic status in depressed participants in comparison with normal subjects, which was consistent with other research [47, 48]. In this regard, a cross-sectional study of 4,933 older adults in China reported a positive association between lower education level (less than 6 y) and monthly household income and late-life depression [47]. Generally, socioeconomically disadvantaged older adults may have greater exposure to stressors, which negatively affect their mental health [47].

According to our findings, older adults who lived alone were more likely to be depressed compared to those who lived with their family/nurse, which is also supported by previous research [47, 49, 50]. Living alone may result in social isolation, which has been linked with moderate and severe depression in older adults [51]. Furthermore, a higher percentage of depressed seniors in the present study reported gastrointestinal or oral disorders. In line with this finding, previous research revealed a positive association between gastrointestinal symptoms [52] or oral health outcomes [53] and depression in older adults. The gut-brain axis may involve in the bi-directional communication between depression and gastrointestinal symptoms [52]. Moreover, visceral hypersensitivity and altered perception of esophageal stimuli in patients with depression may contribute to several gastrointestinal symptoms [52].

We discovered that seniors with a higher BMI were more likely to experience depression than those with a lower BMI. Arigo D et al. [54] found a similar result from a longitudinal study recruiting 5688 middle-aged and older adults, indicating that higher BMI was correlated with higher depressive symptoms. In the current research, older adults with irregular sleep patterns were more likely to suffer from depression. Similarly, Pye J et al. [55] found a significant negative relationship between Sleep Regularity Index (SRI) and depression among 138 older adults in Australia. Evidence reveals a close relationship between poor sleep and aging-related cognitive impairment, which could be a reason for depression [56].

Few studies have investigated the relationship between diet quality and depression in older adults, and the present study is the first one in the Middle East region. Nevertheless, a number of studies have been published assessing the association between geriatric nutritional status or their dietary intake pattern and depression in the Middle East and developing countries. A previous experimental study conducted in a family health center over 356 older adults in Egypt reported a significant association between nutritional status and depression (*p* < 0.01) [57]. Another cross-sectional study of 400 seniors in Bangladesh found a significantly higher risk of depression for participants suffering from malnutrition in comparison with those having normal nutritional status (*p* < 0.001) [41]. Consistently, a recent study of 116 Jordanian seniors has shown that depressive symptoms were significantly higher among seniors following an unhealthy dietary pattern [58].

In this study, older adults in the highest tertile of HEI-2015 had a lower chance of depression. As with our result, several studies have demonstrated that seniors with lower nutritional quality are more likely to experience depressive symptoms. A systematic review and meta-analysis (2018) on healthy dietary indices and risk of depressive outcomes showed an inverse relationship between the Healthy Eating Index-2005 (HEI-2005) or Alternative Healthy Eating Index-2010 (AHEI-2010) and the prevalence of depression in cross-sectional studies on adults [5]. A population-based cross-sectional study among 820 Brazilian adults and older adults, participants with the worst diet quality had a higher chance of experiencing major depressive episodes [59]. Another cross-sectional study on 3363 Iranian adults has shown that higher adherence to the Adjusted Healthy Eating Index (AHEI-2010) was associated with decreased odds of depression and anxiety in women and participants who were 40 years old or younger [20]. Similar findings were reported from a cohort study in 15,093 adults in Spain that revealed a significant inverse association between AHEI-2010 and depression risk after a mean of 8.5 years of follow-up [60]. Moreover, a longitudinal study of 26,225 participants aged 18–86 y in France, 2166 incident cases of depressive symptoms were identified and a significant inverse association was found between the Probability of Adequate Nutrient Intake Dietary Score (PANDiet) and Diet Quality Index-International (DQI-I) over an average of 6 years follow-up; However, they found no similar significant relationship between AHEI-2010 and depressive symptoms [61].

After adjustment for different confounders, we did not discover a significant relationship between HEI components and depression in older adults, however previous studies revealed a significant association between subscales of indexes measuring the quality of diet and depression. Beydoun et al. [62] have found a significant inverse association between some of the HEI-2005 components and depressive symptoms among women, indicating that higher intake of total vegetables, dark green and orange vegetables, meat and beans, and a lower intake of discretionary fat, alcohol, and added sugars were significantly associated with lower CES-D scores.

A number of biological mechanisms have been suggested to explain the association between diet quality and mental health. A recent meta-analysis found a possible role of diet quality on systemic inflammation and the reduction of neuronal damage due to oxidative stress that can affect depression and mental health [5]. Also, it has been shown that diet quality can play a role in mood and behavioral disorders by affecting the gastrointestinal microbiota [63]. Moreover, diet may affect depression by altering the pathways involved in mitochondrial dysfunction, tryptophan–kynurenine metabolism, and obesity [64]. Several interconnected pathways have been reported to explain the relationship between diet, mood disorders, and obesity including reduced serotonin and dopamine levels and excessive secretion of glucocorticoids [65]. Further research is needed to explain plausible mechanisms of the association between diet quality and depression.

Some evidence has indicated that there is a two-way correlation between depression and diet quality in older adults [66]. Depression may lead to lower quality and diversity of diet; for instance, Jacka et al. [67] suggested that individuals with current depression are more likely to have poorer dietary habits, which is probably due to the calmative effects of antidepressant drugs in the short term and their noxious effects in the long term. However, due to the cross-sectional nature of the present study, it is not possible to determine whether seniors eat poorly since they are depressed and do not care about their health or since they eat poorly, they have become susceptible for depression. Nevertheless, findings from a comprehensive systematic review suggested that overall diet quality may play an important role as a potentially modifiable risk factor which may cover all programs regarding prevention and control of depression [68].

The main strength of the present study was that it focused on community-living older adults instead of institutionalized seniors. Moreover, sampling from all geographic regions of Tehran city in various settings (health centers, mosques, and *Saraye Mahalleh*) made the data generalizable to the entire senior community of Tehran city. In addition, the reasonable response rate (80%) increased the generalizability, as did the adjustment of various variables to explore the much more precise association between diet quality and depression in the analysis. However, in evaluating the current study, several limitations should be considered. First, the causality of the relationship between depression and diet quality in older adults couldn’t be inferred because of the cross-sectional design of the study. Second, in evaluating dietary intake of individuals through 24-h recalls, some sources of bias existed as some of the seniors weren’t able to remember what they had eaten the day prior to the interview. This problem was more serious with regard to mixed dishes and their ingredients and salt intake in men. In order to reduce this recall bias, their dietary intake information was double checked with their spouse or one of their family members who was in close contact.

## Conclusion

Findings suggested that diet quality may be associated with depression in the studied free-living older adults. Due to the uncertainty of results, further studies with a stronger design, such as cohort studies, are needed to clarify the possible causal relationship in the senior population.

Considering the notable prevalence of depression in older adults, especially women, and its higher possibility in those with low quality of diet, especially in the group with lower socio-economic status, designing interventional programs or financial and educational policies to improve the quality of the seniors’ diet as well as their mental condition seems to be necessary.

## Supplementary Information

**Additional file 1: Table S1. **The Odds ratio and *95% Confidence Interval (CI) *of independent and confounding variables included in the first adjusted logistic model. **Table S2.** The Odds ratio and *95% Confidence Interval (CI) *of independent and confounding variables adjusted in the second logistic model. **Table S3.** The Odds ratio and *95% Confidence Interval (CI) *of independent and confounding variables considered in the third logistic model.

## Data Availability

The datasets obtained and/or analyzed during the current study are not publicly available as the datasets are highly detailed and we are planning to publish more papers using the same dataset but are available from the corresponding author upon reasonable request.

## Abbreviations

ESRD: End Stage Renal Disease
USDA: United States Department of Agriculture
FCT: Food Composition Table
HEI: Healthy Eating Index
GDS: Geriatric Depression Scale
ADL: Activity of Daily Living
IADL: Instrumental Activity of Daily Living
SRI: Sleep Regularity Index
AHEI: Alternative Healthy Eating Index
PANDiet: Probability of Adequate Nutrient Intake Dietary Score
DQI-I: Diet Quality Index-International
CES-D: Center for Epidemiologic Studies-Depression
