# Supplementary material for: Relationship between diet quality and depression among Iranian older adults in Tehran
Source: BMC Geriatr. 2022 Aug 26;22:708. doi: 10.1186/s12877-022-03380-1 (PMC9419352; doi:10.1186/s12877-022-03380-1)
Supplement: Supplementary file 1 — Additional file 1: Table S1. The Odds ratio and 95% Confidence Interval (CI) of independent and confounding variables included in the first adjusted logistic model. Table S2. The Odds ratio and 95% Confidence Interval (CI) of independent and confounding variables adjusted in the second logistic model. Table S3. The Odds ratio and 95% Confidence Interval (CI) of independent and confounding variables considered in the third logistic model. [file 12877_2022_3380_MOESM1_ESM.docx]

**Table S1.** The Odds ratio and ***95% Confidence Interval (CI)*** of independent and confounding variables included in the first adjusted logistic model.

| Depression | | | |
| --- | --- | --- | --- |
|  | *OR* | ***95% CI*** | ***P-value*** |
| Tertiles of HEI-2015 score |  |  |  |
| T1 | 1.000 | (*Ref.*) |  |
| T2 | 0.690 | (0.163 – 2.916) | **0.614** |
| T3 | 0.418 | (0.089 – 1.969) | **0.270** |
| Age (years) | 1.037 | (0.731 – 1.471) | **0.841** |
| Sex  Male  Female | 1.000  1.756 | *(Ref.)*  (1.014 – 3.042) |  |
|  |  |  |  |
|  |  |  | **0.044** |
| Marital status  Single/divorced/widow  Married | 1.000  0.761 | *(Ref.)*  (0.423 – 1.370) |  |
|  |  |  |  |
|  |  |  | **0.363** |
| ADL | 0.546 | (0.212 – 1.403) | **0.209** |
| IADL | 1.906 | (1.186 – 3.065) | **0.008** |
| Energy intake (kcal/day) | 0.999 | (0.998 – 0.999) | **<0.001** |
| HEI-2015 ^a^ | 0.621 | (0.348 – 1.111) | **0.108^b^** |

Depression was considered as a dependent binary variable and HEI as the independent variable.

*ADL* activities of daily living, *IADL* instrumental activities of daily living

^a^ As a basis for the trend test, HEI-2015 score was constructed from the categorized variable and placed into the model as a successive integer.

^b^ p-for trend

**Table S2.** The Odds ratio and ***95% Confidence Interval (CI)*** of independent and confounding variables adjusted in the second logistic model.

| Depression | | | |
| --- | --- | --- | --- |
|  | *OR* | ***95% CI*** | ***P-value*** |
| Tertiles of HEI-2015 score |  |  |  |
| T1 | 1.000 | (*Ref.*) |  |
| T2 | 0.558 | (0.123 – 2.532) | **0.450** |
| T3 | 0.314 | (0.061 – 1.613) | **0.165** |
| Age (years) | 0.974 | (0.668 – 1.418) | **0.890** |
| Sex |  |  |  |
| Male | 1.000 | (*Ref.*) |  |
| Female | 1.695 | (0.941 – 3.050) | **0.079** |
| Marital status |  |  |  |
| Single/divorced/widow | 1.000 | (*Ref.*) |  |
| Married | 1.019 | (0.477 – 2.175) | **0.962** |
| ADL | 0.544 | (0.199 – 1.485) | **0.235** |
| IADL | 1.663 | (0.988 – 2.800) | **0.055** |
| Energy intake (Kcal/day) | 0.999 | (0.999 – 1.000) | **0.002** |
| Education |  |  |  |
| Illiterate/Primary/secondary school/High school diploma | 1.000 | (*Ref.*) |  |
| University degree | 0.698 | (0.438 – 1.111) | **0.130** |
| Living status |  |  |  |
| Alone | 1.000 | (*Ref.*) |  |
| With family/nurse | 0.961 | (0.379 – 2.441) | **0.934** |
| House possession |  |  |  |
| Yes | 1.000 | (*Ref.*) |  |
| No | 1.099 | (0.729 – 1.658) | **0.651** |
| Household income (million IRR) | 0.515 | (0.368 – 0.719) | **<0.001** |
| Receiving food aids |  |  |  |
| No | 1.000 | (*Ref.*) |  |
| Yes | 0.855 | (0.257 – 2.655) | **0.787** |
| Receiving social services |  |  |  |
| No | 1.000 | (*Ref.*) |  |
| Yes | 1.239 | (0.369 – 4.161) | **0.729** |
| Insurance coverage |  |  |  |
| Yes | 1.000 | (*Ref.*) |  |
| No | 1.232 | (0.446 – 3.407) | **0.687** |
| Supplemental insurance coverage |  |  |  |
| Yes | 1.000 | (*Ref.*) |  |
| No | 0.972 | (0.569 – 1.663) | **0.918** |
| HEI-2015 ^a^ | 0.562 | (0.299 – 1.056) | **0.073^b^** |

Depression was considered as a dependent binary variable and HEI as the independent variable.

*ADL* activities of daily living, *IADL* instrumental activities of daily living

^a^ As a basis for the trend test, HEI-2015 score was constructed from the categorized variable and placed into the model as a successive integer.

^b^ p-for trend.

**Table S3.** The Odds ratio and ***95% Confidence Interval (CI)*** of independent and confounding variables considered in the third logistic model.

| Depression | | | |
| --- | --- | --- | --- |
|  | *OR* | ***95% CI*** | ***P-value*** |
| Tertiles of HEI-2015 score |  |  |  |
| T1 | 1.000 | (*Ref.*) |  |
| T2 | 0.429 | (0.057 – 3.205) | **0.409** |
| T3 | 0.176 | (0.020 – 1.524) | **0.115** |
| Age (years) | 0.976 | (0.624 – 1.527) | **0.915** |
| Sex |  |  |  |
| Male | 1.000 | (*Ref.*) |  |
| Female | 1.649 | (0.703 – 3.869) | **0.250** |
| Marital status |  |  |  |
| Single/divorced/widow | 1.000 | (*Ref.*) |  |
| Married | 0.968 | (0.390 – 2.399) | **0.944** |
| ADL | 0.503 | (0.154 – 1.638) | **0.254** |
| IADL | 1.040 | (0.547 – 1.980) | **0.904** |
| Energy intake (Kcal/day) | 0.999 | (0.999 – 1.000) | **0.005** |
| Education |  |  |  |
| Illiterate/Primary/secondary school/High school diploma | 1.000 | (*Ref.*) |  |
| University degree | 0.692 | (0.400 – 1.200) | **0.190** |
| Living status |  |  |  |
| Alone | 1.000 | (*Ref.*) |  |
| With family/nurse | 2.036 | (0.597 – 6.944) | **0.256** |
| House possession |  |  |  |
| Yes | 1.000 | (*Ref.*) |  |
| No | 0.928 | (0.569 – 1.513) | **0.764** |
| Household income (million IRR) | 0.509 | (0.343 – 0.756) | **0.001** |
| Receiving food aids |  |  |  |
| No | 1.000 | (*Ref.*) |  |
| Yes | 1.045 | (0.268 – 4.071) | **0.949** |
| Receiving social services |  |  |  |
| No | 1.000 | (*Ref.*) |  |
| Yes | 1.762 | (0.446 – 6.961) | **0.419** |
| Insurance coverage |  |  |  |
| Yes | 1.000 | (*Ref.*) |  |
| No | 1.216 | (0.363 – 4.069) | **0.751** |
| Supplemental insurance coverage |  |  |  |
| Yes | 1.000 | (*Ref.*) |  |
| No | 0.975 | (0.508 – 1.871) | **0.939** |
| Having gastrointestinal problems |  |  |  |
| Yes | 1.000 | (*Ref.*) |  |
| No | 0.722 | (0.361 – 1.446) | **0.358** |
| Loss of appetite |  |  |  |
| Yes | 1.000 | (*Ref.*) |  |
| No | 0.337 | (0.127 – 0.894) | **0.029** |
| Dysphagia |  |  |  |
| Yes | 1.000 | (*Ref.*) |  |
| No | 0.160 | (0.038 – 0.674) | **0.013** |
| Dry mouth |  |  |  |
| Yes | 1.000 | (*Ref.*) |  |
| No | 1.595 | (0.733 – 3.470) | **0.239** |
| Maldigestion |  |  |  |
| Yes | 1.000 | (*Ref.*) |  |
| No | 1.689 | (0.648 – 4.405) | **0.283** |
| Diarrhea |  |  |  |
| Yes | 1.000 | (*Ref.*) |  |
| No | 6.066 | (0.633 – 58.147) | **0.118** |
| Constipation |  |  |  |
| Yes | 1.000 | (*Ref.*) |  |
| No | 1.240 | (0.609 – 2.523) | **0.553** |
| Chewing difficulty |  |  |  |
| Yes | 1.000 | (*Ref.*) |  |
| No | 0.318 | (0.141 – 0.717) | **0.006** |
| Oral problems |  |  |  |
| Yes | 1.000 | (*Ref.*) |  |
| No | 0.846 | (0.440 – 1.627) | **0.617** |
| Taking Medication |  |  |  |
| Yes | 1.000 | (*Ref.*) |  |
| No | 0.439 | (0.134 – 1.442) | **0.175** |
| Diabetes medications |  |  |  |
| Yes | 1.000 | (*Ref.*) |  |
| No | 1.379 | (0.717 – 2.650) | **0.336** |
| Hyperlipidemia medications |  |  |  |
| Yes | 1.000 | (*Ref.*) |  |
| No | 1.132 | (0.607 – 2.113) | **0.696** |
| Hypertension medications |  |  |  |
| Yes | 1.000 | (*Ref.*) |  |
| No | 0.815 | (0.427 – 1.557) | **0.536** |
| Cardiovascular medications |  |  |  |
| Yes | 1.000 | (*Ref.*) |  |
| No | 0.840 | (0.422 – 1.674) | **0.621** |
| Dietary supplement intake |  |  |  |
| Yes | 1.000 | (*Ref.*) |  |
| No | 1.121 | (0.564 – 2.227) | **0.745** |
| Currently smoking |  |  |  |
| No | 1.000 | (*Ref.*) |  |
| Yes | 0.946 | (0.312 – 2.870) | **0.922** |
| Sleep habits |  |  |  |
| Regular | 1.000 | (*Ref.*) |  |
| Irregular | 3.106 | (1.692 – 5.700) | **<0.001** |
| Weight (gram) | 1.023 | (0.975 – 1.073) | **0.360** |
| Waist circumference (mm) | 1.006 | (0.965 – 1.050) | **0.767** |
| Mid-arm circumference (mm) | 0.944 | (0.830 – 1.073) | **0.380** |
| Calf circumference (mm) | 0.986 | (0.889 – 1.093) | **0.787** |
| Obesity |  |  |  |
| No | 1.000 | (*Ref.*) |  |
| Yes | 0.994 | (0.416 – 2.379) | **0.990** |
| HEI-2015 ^a^ | 0.414 | (0.195 – 0.875) | **0.021^b^** |

Depression was considered as a dependent binary variable and HEI as the independent variable.

*ADL* activities of daily living, *IADL* instrumental activities of daily living

^a^ As a basis for the trend test, HEI-2015 score was constructed from the categorized variable and placed into the model as a successive integer.

^b^ p-for trend
